# Variations in Heat Load Nutritional Management on Animal Performance, Rumen Temperature and pH Characteristics in Grain-Fed Steers Challenged by High Heat Load

**DOI:** 10.3390/ani15243615

**Published:** 2025-12-15

**Authors:** Stephanie L. Sammes, Grace P. James, Megan L. Sullivan, Allan T. Lisle, Angela M. Lees, Gene Wijffels, John B. Gaughan

**Affiliations:** 1 Animal Science Group, School of Agriculture and Food Sustainability, The University of Queensland (Gatton Campus), Gatton, QLD 4343, Australia; steph.sammes@feedworks.com.au (S.L.S.); j.gaughan@uq.edu.au (J.B.G.); 2 FeedWorks Australia, Lancefield, VIC 3435, Australia; 3 Nutrition Service Associates, Toowoomba, QLD 4350, Australia; 4 CSIRO Agriculture and Food, Queensland Bioscience Precinct, St. Lucia, Brisbane, QLD 4067, Australia; gene.wijffels@csiro.au

**Keywords:** body temperature, core body temperature, feedlot, heat stress, heat wave, rumen physiology, temperature humidity index

## Abstract

This study evaluated the effects of including additional roughage and the timing of roughage addition on rumen temperature (T_RUM_), rumen pH, dry matter intake as a percentage of live weight (DMILW), water consumption as a percentage of live weight (WILW) and average daily gain of grain-fed steers exposed to a simulated heat wave. A total of forty-eight Black Angus steers (539.53 ± 4.95 kg) were housed within climate control rooms for 21 days and exposed to a 5-day simulated heat wave. Steers were randomly allocated into four cohorts with 12 steers/cohort, and then allocated to one of three dietary treatments: Treatment 1 (T1) were fed a finisher diet for the 21 days; Treatment 2 (T2) transitioned from the finisher diet to a heat load diet on d 9 and fed the heat load diet until d 14; and Treatment 3 (T3) transitioned from the finisher diet to the heat load diet on d 7 and fed the heat load diet until d 14. On d 15, T2 and T3 transitioned back to the finisher diet. The study was categorised into five phases consisting of (i) Phase I, d 0–6 (Temperature Humidity Index, THI 65 to 78); (ii) Phase II, d 7–8 (THI 65 to 78); (iii) Phase III, d 9–11 (THI 83 to 90); (iv) Phase IV, d 12–13 (THI 78 to 85); and (v) Phase V, d 14–20 (THI 65 to 78). During the heat wave challenge in Phase III, all Treatments exhibited lower DMILW (*p* < 0.0001), greater T_RUM_ and rumen pH (*p* < 0.0001), lower ranges in T_RUM_ and rumen pH (*p* ≤ 0.0005) and altered diurnal T_RUM_ and rumen pH rhythms. Average daily gain was not influenced by Treatments (*p* ≥ 0.98). Overall, these results suggest that nutritional management remains an important consideration to reduce the impact of hot climatic conditions on the rumen environment during heat wave and post-heat wave conditions.

## Simple Summary

Globally, heat load is well documented to have a negative influence on the well-being and productivity of feedlot cattle. Despite considerable research advancements, the management of feedlot cattle during hot conditions remains challenging. Nutritional management strategies present a targeted pathway to support feedlot cattle during these periods. In this study, three feeding strategies were implemented during a simulated heat wave event. The climatic conditions imposed on these cattle were sufficient to elicit heat load responses, allowing for the effectiveness of the three feeding strategies to be evaluated. All cattle experienced significant physiological changes, as indicated by alterations in feed and water intake, rumen temperature and rumen pH and their diurnal rhythms. The outcomes from this study highlight that the nutritional management of feedlot cattle is an important aspect for maintaining the rumen environment, which may in turn facilitate maintained animal health and performance. Furthermore, this study highlighted that feeding strategies to alleviate the negative impacts on the rumen environment may be more important to implement post-heat wave, rather than prior to or during heat wave conditions.

## 1. Introduction

Globally, heat stress during the summer months constitutes a major welfare and production challenge [1]. During the summer months, when climatic conditions can be categorised as chronic heat load, feedlot cattle are also often exposed to heat wave events that represent a significant risk period for these animals [2,3,4,5]. The animal factors and environmental conditions that are associated with feedlot cattle responses to heat load have been well established [6,7,8]. One well documented response is a reduction in dry matter intake [9,10,11]. Voluntary reductions in dry matter intake are associated with a reduction in metabolic heat production, and while this is a good survival strategy it can adversely affect rumen function and overall animal performance [12]. Specifically, when DMI is reduced, or feed consumption is variable, disturbances in rumen pH can occur, subsequently increasing the risk of rumen dysfunction and metabolic disorders, such as acidosis [13,14,15,16,17]. This risk is heightened in feedlot cattle as they are fed high energy and highly fermentable grain-based rations that contribute to a high metabolic heat load [18].

Nutritional management strategies specifically targeted for supporting feedlot cattle during heat load may present a beneficial and cost-effective strategy to support cattle health and well-being during these conditions. Previous research investigating nutritional management strategies include modifying the timing of feeding [19,20], restricting feed intake [9,20,21] and changing diet composition [22] to maintain animal performance and support rumen health of feedlot cattle during heat wave events. Anecdotally, feeding strategies, including timing of feeding and changing dietary composition, are approaches utilised by commercial feedlots in Australia’s feedlot industry as a heat load management tool. However, there is limited literature describing the implementation of specific heat load rations for grain-fed cattle during heat wave conditions.

Therefore, the objective of this study was to determine the effect of different feeding strategies, and time of feeding on rumen temperature (T_RUM_), rumen pH, dry matter intake as a percentage of live weight (DMILW), water intake as a percentage of live weight (WILW) and average daily gain of grain-fed steers exposed to a simulated acute heat wave event. It was hypothesised that feeding cattle a heat load diet for three days before a simulated heat wave would help maintain T_RUM_ and rumen pH, thereby minimising the performance losses, when compared with implementing a heat load diet at the onset of the heat wave or feeding a finisher diet during heat wave conditions.

## 2. Materials and Methods

The study was undertaken at The University of Queensland’s large animal research facility, Queensland Animal Science Precinct, located in Southeast Queensland, Australia (27.54° S, 152.34° E; 100 m above mean sea level) during a southern hemisphere summer (December to April).

### 2.1. Animals and Animal Management

A total of 48 purebred yearling Black Angus steers with an initial non-fasted live weight of 539.53 ± 4.95 kg were used in a 21-day climate control study. Steers were randomly allocated into four cohorts consisting of 12 steers per cohort. Steers were randomly allocated to three dietary treatments (described below), thus there were four steers per treatment within each of the four cohorts. Steers in each cohort were group-housed (*n* = 12) in singular feedlot pens (162 m^2^, 27 m × 6 m) per cohort for 50 days, prior to entry into the climate control chambers. Steers were relocated from group-housed feedlot pens to individual pens (30 m^2^, 3 × 10 m) on d −10 and housed in individual pens for the 10 days directly prior to entry into the climate control chambers, respectively, for each of the four cohorts.

Animal observational data were collected on each individual steer at 2 h intervals between 6:00 am and 6:00 pm on days 3, 4, 6, 7 and d 8; and on days 13, 14, 15, 16, 18, 19 and 20 (data not reported here). Observational data were then obtained at 1 h intervals over 24 h (from 12:00 am to 11:00 pm, daily) on day 5, during the simulated heat wave event over days 9 to 13 and on day 17 (data not reported here). At each observation, cattle were observed for panting score; respiration rate (RR); posture (standing/lying); and activity (eating/drinking/ruminating) (data not reported here).

Individual steer non-fasted liveweight was obtained using calibrated scales and load beams (Gallagher, Melbourne, Australia) on entry into the climate control chambers (d 0) at 7:00 am and on exit (d 20) at 6:30 pm, with individual steer average daily gain (kg/steer/d) determined. On enrolment into this study and entry to the climate control chambers, steers were 60 days on feed.

Steers were vaccinated for clostridial diseases (enterotoxaemia, tetanus, blacks disease, malignant oedema and blackleg; Ultravac 5 in1; Pfizer Animal Health, Sydney, Australia), bovine respiratory disease (Bovillis MH, in-activated *Mannheimia haemolytica*; Coopers Animal Health, Sydney, Australia) and were treated for internal and external parasites (Cydectin, Fort Dodge Australia, Baulkham Hills, NSW, Australia) 10 days prior to feedlot entry, 69 days prior to the commencement of this study. Steers received a secondary bovine respiratory disease vaccination on d −46 (Bovillis MH, in-activated *Mannheimia haemolytica*; Coopers Animal Health, Australia). All steers were implanted with a hormonal growth promotant on d −60 (Synovex^®^ Plus, 200 mg trenbolone acetate + 28 mg estradiol benzoate, Zoetis, Sydney, Australia).

Steers were part of a larger 165-day study (data not presented here). On exit from the climate control chambers, all steers were group-housed (*n* = 12) in a singular feedlot pen (162 m^2^, 27 m × 6 m) for 22 days until slaughter at a commercial abattoir.

### 2.2. Climate Control Chambers

Two climate control chambers were using in this study. The climate control chambers were configured in such a way that there were three individual animal pens (6.25 m^2^; 2.5 × 2.5 m) on each side of the climate control chamber, thus housing 6 steers per chamber. Each individual animal pen was constructed of four metal cattle panels and were constructed over a grated platform floor, allowing for easy cleaning and drainage. The grated flooring was covered with a rubber mat (SureFoot^®^ Mat, 1.22 m × 1.83 m, 1.8 cm thickness; RPS Industries Australia, Melbourne, Australia) to improve animal comfort. The pen design did not restrict animal movement, steers were able to freely turn around, lay down, self-groom and have visual contact with conspecifics housed within the same chamber, but were unable to physically touch their conspecifics.

Each steer was provided with individual ad libitum access to a water trough (700 mm length × 500 mm width × 280 mm depth) fitted with an inline water metre (RMC Zenner, Melbourne, Australia). Steers also had individual access to a feed trough (500 mm length × 500 mm width × 500 mm depth). The climate control chambers were cleaned daily at 0830 h prior to feeding (described below) by hosing all excrement from the mats and pen flooring into the drainage system below the grated flooring.

### 2.3. Climatic Conditions

Steers were exposed to a simulated Australian summer that was established from 5 years of historical weather data (Dalby, Qld; 27.19° S, 151.27° E), which included the minimum, maximum and mean data for ambient temperature (°C) and relative humidity (%), obtained from the Australian Bureau of Meteorology (www.bom.gov.au). From these data, the temperature humidity index (THI) was calculated using the equation below, as modified from Thom [23]:

THI = [0.8 × T_A_] + [(% RH ÷ 100) × (T_A_ − 14.4)] + 46.4

Climatic data was used to create a climate schedule to simulate both average summer and a 5-day heat wave event throughout the 21-day study. The study was split into five Phases consisting of pre-heat wave conditions (i) Phase I and Phase II, where the THI ranged between 65 and 78, ambient temperature was maintained between 19 and 31 °C and RH was held between 40 and 80%, over the period encompassing d 0 to d 8. Phase III imposed the acute heat wave conditions where the THI ranged between 83 and 90, ambient temperature was maintained between 30 and 40 °C, with relative humidity held between 45 and 80%, over d 9 to d 11. Imposed ambient conditions then decreased slightly during Phase IV, where the THI ranged between 78 and 85, where ambient temperature was maintained between 28 and 36 °C and relative humidity held between 45 and 70%, over d 12 and d 13. During the post-heat wave conditions, Phase V, the ambient conditions returned to baseline summer conditions, where the THI ranged between 65 and 78, ambient temperature was maintained between 19 and 31 °C and relative humidity was held between 40 and 80%, over d 14 until exit from the climate control chambers on d 20.

The climate schedule during Phase I and Phase II followed a diurnal pattern reaching a maximum of 31 °C, 40% RH, resulting in a corresponding THI of 78 during daytime hours (6:00 am to 6:00 pm) and a maximum 19 °C, 80% RH, and a THI of 65 during nighttime hours (6:00 pm to 6:00 am). During Phase III, during the heat wave between d 9 and d 11, climatic conditions during daytime hours reached a maximum 40 °C, 45% RH, and a THI of 90, and a corresponding nighttime maximum of 30 °C, 80% RH, imposing a THI of 83. Throughout Phase IV, covering d 12 and d 13, climatic conditions imposed consisted of a maximum 36 °C, 45% RH, resulting in a THI of 85 during daytime hours, and a maximum 28 °C, 70% RH, and a THI of 78 during nighttime hours. The climate regime for Phase V was scheduled to be the same conditions as those imposed during Phase I and Phase II as described above. The lighting schedule involved 100% lighting from 5:01 am to 6:59 pm and 10% lighting (moonlight) from 7:00 pm to 5:00 am daily.

### 2.4. Nutritional Management

The steers were fed twice daily at approximately 9:00 am and 1:00 pm with 50% of the ration provided at each feed offering. At 8:30 am, immediately prior to the 9:00 am feeding, any feed refusals were removed and weighed, these data were used to determine daily as fed feed intake (kg/steer/d) and dry matter intake (kg/steer/d) based on dry matter % of the diet from dietary analysis (Table 1). Individual feed allocation was allocated based on each individuals’ feed consumption from the day prior and managed throughout the duration of the study, with careful consideration during the heat wave challenge during Phase III between day 9 and 11. Using individual steer dry matter intake and liveweight data from d 0, the dry matter intake as a proportion of LW was determined (DMILW; % LW/steer/d). Water metre readings were recorded daily at 6:00 am; from these, data individual water intake (L/steer/d) and water intake as a proportion of liveweight, obtained from d 0, were determined (WILW; % LW/steer/d). All water troughs were cleaned, emptied and refilled as required. The total water removed and refilled for each cleaning event from each individual water trough was subtracted from the daily water intake for each respective steer.

Each cohort was commenced on a dietary transition protocol that allowed for the transition from a pasture-based diet to feedlot finisher ration while each respective cohort was housed as a group in feedlot pens during the 60 days prior to feedlot entry. Steers commenced on a starter ration on entry to the feedlot on d −60, were transitioned to an intermediate ration on d −53 and then to a finisher diet on d −46 (Table 1). While housed in the climate control chamber, three dietary management treatments were implemented: Treatment 1 (T1) cattle were fed a finisher diet for the 21 days; Treatment 2 (T2) cattle were transitioned from the finisher diet to a heat load diet (Table 1) on d 9 and remained on this diet until d 14; and Treatment 3 (T3) cattle were transitioned from the finisher diet to the heat load diet on d 7 and remained on this diet until d 14 (T3). Steers that were transitioned onto the heat load diet in T2 and T3 commenced transitioning back to the standard finisher diet on d 15. This transition to the standard finisher diet occurred using a step-up protocol where on d 15, T2 and T3 were fed 70% heat load diet and 30% finisher diet, then on d 16 and d 17 steers were 50% heat load diet and 50% finisher diet, then returning to 100% finisher diet on d 18.

Dietary formulation was influenced by ingredient availability. Lucerne hay was used to increase the roughage proportion of the heat load diet as this was readily available and cost-effective. Diets were prepared on site at one-month intervals in a commercial grade mixing wagon (Kuhn Euromix II 1860, Saverne, France). Because of this, ingredients needed to be sourced so that dry diets could be formulated and easily stored until use between mixings. A 500 g grab sample was collected from each diet mixing by collecting sub-samples of the mixed sections. Diet samples were frozen at −20 °C until analysis, which was conducted at the end of the larger study experimental period. Each dietary sample was analysed by a commercial analytical laboratory (Symbio Laboratories, Brisbane, OLD, Australia, Table 1).

### 2.5. Rumen Temperature and Rumen pH

Steers were orally administered with an active radio frequency identification (RFID) transmitting rumen bolus (smaXtec pH Plus Bolus, smaXtec, Graz, Austria) on d −10. Individual boluses were cylindrical in shape (132 mm length × 35 mm diameter) and weighed approximately 220 g. Prior to administration, the smaXtec boluses were checked for temperature stability by placing them in a 39 °C water bath for 24 h. Prior to this, rumen boluses were calibrated by placing boluses in a pH 4 solution for 8 h and then another 8 h in a pH 7 solution, as per manufacturer specifications. Rumen temperatures and ruminal pH were recorded at 10 min intervals, from 24 h after they were orally administered. These data were communicated real-time to a base station (smaXtec Base Station, smaXtec, Austria) before transmission to a data server and storage in an online data base (smaXtec Messenger, smaXtec, Austria). Rumen boluses were recovered from all cattle post-slaughter.

### 2.6. Statistical Analysis

Six steers were removed from study during the first 2 days of Phase III due to an adverse response to conditions; specifically, steers exhibited a combination of highly agitated/distressed behaviours (sunken eyes, lack lustre appearance, vocalisation), T_RUM_ > 42 °C, RR > 160 bpm, panting score ≥ 3.5, acute acidosis symptoms, hypersalivation, rumen pH > 5.0, ceased/very low DMILW and WILW. Three of these steers were from T1 and three were from T2; as such, data from these steers was excluded from statistical analysis.

All data were analysed using R3.6.2 software (R, R Foundation for Statistical Computing, Vienna, Austria). Day 0 and d 1 was used as an opportunity for all the steers to acclimate to the climate control chamber, and as such, data during this time was omitted. In addition, d 20 was also omitted.

Each day was considered to go over a 24 h period that encompassed the period between 6:00 am and 5:59 am to coincide with behavioural observational data collection (data not presented here).

Days of the study were then split into the five Phases for comparison of animal data based on transition to diets and climate schedule as previously described.

Rumen variables were investigated as duration of time (DUR, h/d) and magnitude (AUC, area under the curve; AAC, area above the curve) above T_RUM_ and below rumen pH thresholds of biological importance (described below). The AUC and AAC were estimated using the Trapezoid Rule. Rumen temperature variables were calculated above the 42 °C body temperature threshold at which homeostatic systems within the body reach their upper critical limits for normal function, as described by Mehla et al. [24]. From this, T_RUM_ variables included DUR > 42 °C and AAC. Rumen pH variable thresholds of 5.0 and 5.6 were assigned based on work by Nagaraja and Titgemeyer [25], where rumen pH below 5.0 was considered to be acutely acidotic and between 5.0 and 5.6 were considered to be sub-acutely acidotic. From this, rumen pH variables included DUR and AUC below acute rumen acidosis threshold (ARA; pH < 5.0, h/d), and DUR and AUC within subacute rumen acidosis range (SARA; pH < 5.6, ≥ 5.0, h/d). The rumen pH AUC for SARA and ARA, as well as AAC for T_RUM_ DUR > 42 °C, were calculated for each individual animal ID and then averaged values were used for comparisons across Treatment, Day and Phase.

For T_RUM_ and rumen pH data, a linear mixed effects model with a first order auto-regressive error structure was used with fixed effects for pen, cohort, Treatment, Phase, Day, grouped by interaction including Treatment × Phase, Treatment × Day and Treatment × Phase × Day, with animal ID incorporated as a random effect. Range in T_RUM_ and rumen pH was calculated as mean differences between hourly maximum and minimum for each steer within each Treatment.

Animal live weight and average daily gain data were analysed using a mixed effects model that considered pen, cohort and Treatment as the fixed effects and animal ID incorporated as a random effect. Liveweight for DMILW and WILW was determined using the climate chamber entry weight recorded on d 0 for each individual steer. For DMILW and WILW, a linear mixed effects model with a first order ante-dependence covariance structure was used with fixed effects for pen, cohort, Treatment, Phase, Day, grouped by interaction including Treatment × Phase, Treatment × Day and Treatment × Phase × Day, with animal ID incorporated as a random effect.

The DMILW were calculated for each steer using the following equation:

$$DMILW\;(\%\;LW/steer/d)=\frac{DMI\;(kg)\times 100\;}{CCR\;entry\;(d\;0)\;LW}$$

The WILW was calculated for each steer using the following equation:

$$WILW\;(\%\;LW/steer/1/d)=\frac{water\;intake\;(L)\times 100\;}{CCR\;entry\;(d\;0)\;LW}$$

The ADG was calculated for each steer using the following equation:

$$ADG\;(kg/steer/d)=\frac{d\;20\;LW\;(kg)-d\;0\;LW\;(kg)}{20\;(number\;of\;days\;between\;weighings)}$$

Denominator degrees of freedom for statistical tests were calculated using the Kenward–Roger method. Mean values are termed significantly different where *p* ≤ 0.05.

## 3. Results

### 3.1. Temperature Humidity Index

**Figure 1:**
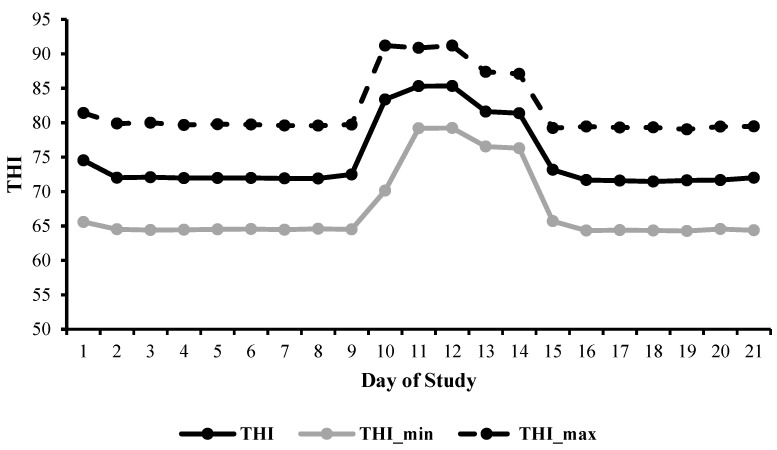
Mean Temperature Humidity Index (THI, solid black line), minimum Temperature Humidity Index (THI_min, solid grey line) and Temperature Humidity Index maximum (THI_max, dashed black line) over the 21-day period when cattle were housed inside the climate control chambers.

Mean, minimum and maximum THI across the 21 days of the climate chamber periods are presented below, in Figure 1. The simulated heat wave challenge commenced on Day 9 and concluded on Day 13. During the simulated heat wave challenge, daytime hours were categorised within the THI stress threshold of ‘emergency’, where THI ≥ 84, and nighttime hours were held within the ‘alert’ threshold, where THI was maintained above 75 and below 78 to prevent heat dissipation in these cattle.

### 3.2. Dry Matter Intake and Average Daily Gain

Mean DMILW was influenced by Day (*p* < 0.0001), Phase (*p* < 0.0001), Phase × Day (*p* < 0.0001) and Treatment × Phase (*p* = 0.002). There were no effects of Treatment (*p* = 0.47), Treatment × Day (*p* = 0.30), or Treatment × Phase × Day (*p* = 0.97). During Phase I and Phase II, mean DMILW were similar (*p* ≥ 0.13) for Treatment (Table 2). The DMILW for T3 during Phase I and Phase II was similar (*p* = 0.20), which coincided with dietary transition to the heat load diet. There was a notable reduction in DMILW (*p* < 0.0001) across all Treatments with the onset of the acute heat load conditions during Phase III. Mean DMILW decreased during Phase III from Phase II (*p* < 0.0001) for T1 (1.15% LW/steer/d; 66.07% decrease), T2 (1.47% LW/steer/d; 77.67% decrease) and T3 (1.28% LW/steer/d; 69.01% decrease). During Phase III, mean DMILW was similar (*p* ≥ 0.08) between Treatments. During Phase IV, mean DMILW was lower (*p* = 0.03) for T2 compared with T3 and tended to be lower (*p* = 0.11) compared with T1, whereas T1 and T3 were similar (*p* = 0.54). Mean DMILW continued to decline until d 11 when mean minimum DMILW occurred for all Treatments (T1, 0.42 ± 0.09% LW/steer/d; T2, 0.18 ± 0.09% LW/steer/d; T3, 0.34 ± 0.08% LW/steer/d). On d 12, DMILW began to increase across all Treatments, returning to levels comparable to those during Phase I and Phase II by d 19. Despite this, mean DMILW during Phase V was lower (*p* < 0.0001) compared with Phase II for T1 (0.34% LW/steer/d; 22.12% decrease), T2 (0.52% LW/steer/d; 28.92% decrease) and T3 (0.42% LW/steer/d; 24.03% decrease). Mean DMILW were similar (*p* ≥ 0.34) for all Treatments during Phase V. There were no differences (*p* ≥ 0.67) for mean LW between Treatments on entry to climate control chambers on d 0 (T1, 537.13 ± 10.03 kg; T2, 538.91 ± 10.11 kg; T3, 542.55 ± 9.33 kg) or upon exit from the climate control chambers on d 20 (T1, 540.72 ± 9.87 kg; T2, 537.04 ± 9.74 kg; T3, 549.01 ± 9.32 kg).

Average daily gains were not influenced by Treatments (*p* ≥ 0.98), whereas mean average daily gain for the 21 days were 0.14 ± 0.21 kg/steer/d, 0.09 ± 0.20 kg/steer/d and 0.09 ± 0.20 kg/steer/d, for T1, T2 and T3, respectively.

### 3.3. Water Intake

**Figure 2:**
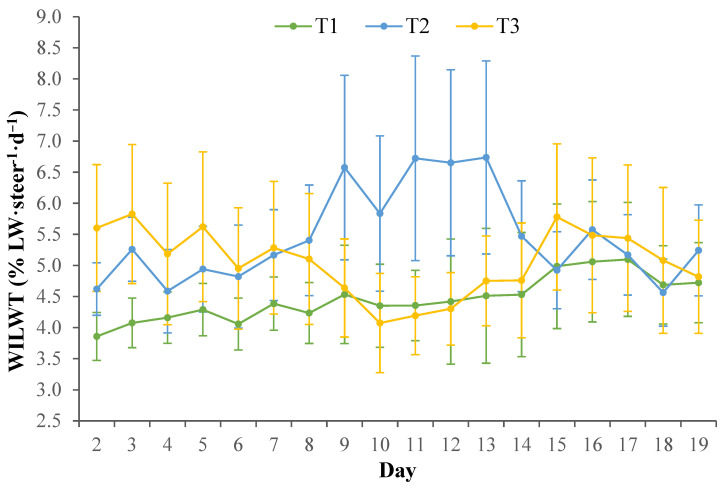
Mean (± SEM) water intake (WI) expressed as a percentage (%) of live weight (WILWT; % LW/steer/d) of steers when housed in climate control rooms for 21 days and split into five PHASE consisting of pre-heat wave conditions (i) d 0–6, Phase I and; (ii) d 7–8, Phase II, to reflect transition to the heat load diet for T3 on d 7; acute heat wave conditions (iii) d 9–11, Phase III and (iv) d 12–13 Phase IV; and post-heat wave conditions (v) d 14–20 Phase V. Three dietary treatments were implemented: Treatment 1 (T1) was fed a finisher diet for the 21 days; Treatment 2 (T2) transitioned from the finisher diet to the heat load diet on d 9 and fed the heat load diet until d 13; Treatment 3 (T3) transitioned from the finisher diet to the heat load diet on d 7 and fed the heat load diet until d 13. On d 14, T2 and T3 transitioned back to the finisher diet.

Mean WILW was influenced by Treatment × Phase (*p* < 0.0001); however, there were no effects of Treatment (*p* = 0.76), Day (*p* = 0.94), Phase (*p* = 0.58), Treatment × Day (*p* = 0.08) or Treatment × Phase × Day (*p* = 1.00). During Phase I and Phase II, mean WILW were similar for all Treatments (*p* ≥ 0.35; Figure 2). The WILW for T3 was similar (*p* = 0.63) on d 6 (4.75 ± 0.94% LW/steer/d) and when transitioned to the heat load diet on d 7 (5.10 ± 0.94% LW/steer/d). Steers in T2 also had similar (*p* = 0.14) WILW on d 8 (5.44 ± 0.99% LW/steer/d) and when transitioned to the heat load diet on d 9 (6.60 ± 0.99% LW/steer/d). When compared with mean WILW during Phase I and Phase II, the mean WILW for T1 during Phase III and Phase IV tended to be greater (*p* ≥ 0.09). Mean WILW for T2 was greater during Phase III (*p* ≤ 0.01) and Phase IV (*p* ≤ 0.03) compared with WILW during Phase I and Phase II (Figure 2). Mean WILW for T3 was greater (*p* ≤ 0.04) during Phase III and Phase IV when compared to Phase I and tended to be greater (*p* ≥ 0.06) when compared with Phase II. During Phase III and Phase IV, the WILW for T2 tended to be greater (*p* ≥ 0.07) compared with T1 and T3. Mean maximum WILW was on d 17 for T1 (5.32 ± 1.00% LW/steer/d), d 13 for T2 (6.79 ± 1.00% LW/steer/d) and on d 3 for T3 (5.62 ± 0.94% LW/steer/d). During Phase V, mean WILW were similar for all Treatments (*p* ≥ 0.89; Figure 2).

### 3.4. Rumen Temperature

#### 3.4.1. Mean Rumen Temperature

**Figure 3:**
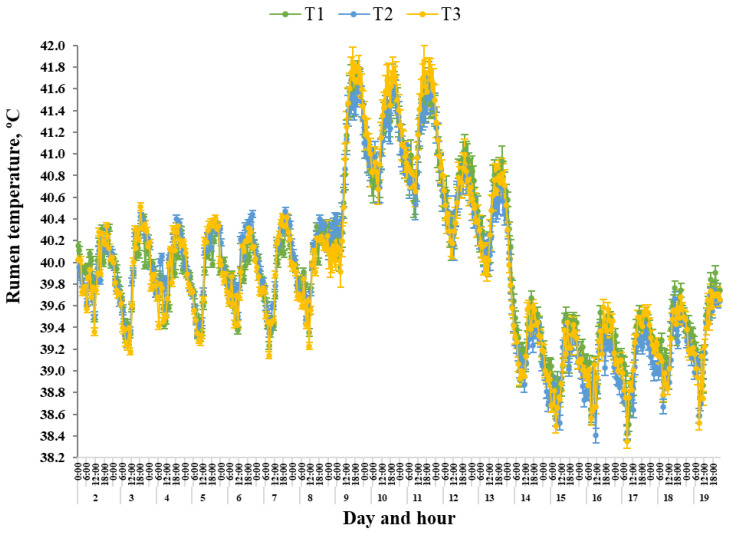
Mean (± SEM) rumen temperature (°C) of steers when housed in climate control rooms for 21 days and split into five Phases consisting of pre-heat wave conditions (i) d 0–6, Phase I and; (ii) d 7–8, Phase II, to reflect transition to heat load diet for T3 on d 7; acute heat wave conditions (iii) d 9–11, Phase III and (iv) d 12–13 Phase IV; and post-heat wave conditions (v) d 14–20 Phase V. Three dietary treatments were implemented: Treatment 1 (T1) was fed a finisher diet for the 21 days; Treatment 2 (T2) transitioned from the finisher diet to a heat load diet (heat load diet) on d 9 and fed the heat load diet until d 13; Treatment 3 (T3) transitioned from the finisher diet to the heat load diet on d 7 and fed the heat load diet until d 13. On d 14, T2 and T3 transitioned back to the finisher diet.

Mean T_RUM_ was influenced by Day (*p* < 0.0001), Phase (*p* < 0.0001) and Phase × Day (*p* < 0.0001). There were no effects of Treatments (*p* = 0.80), Treatment × Day (*p* = 0.99), Treatment × Phase (*p* = 0.12) or Treatment × Phase × Day (*p* = 1.00). Mean T_RUM_ for all Treatments was similar (*p* ≥ 0.93) during Phase I and Phase II (Figure 3). Mean T_RUM_ across the three treatments was lower during Phase IV compared with Phase III (*p* < 0.0001; Figure 3). The mean maximum T_RUM_ was on d 9 for T1 (41.71 ± 0.14 °C), and on d 11 for both T2 (41.65 ± 0.14 °C) and T3 (41.92 ± 0.13 °C). During Phase V, all Treatments had the lowest (*p* < 0.0001) mean T_RUM_ for Phase and was between 0.64 °C and 0.88 °C lower compared with during Phase I and Phase II, 1.86 °C to 2.01 °C lower than Phase III and 1.23 °C to 1.27 °C lower than Phase IV.

#### 3.4.2. Range in Rumen Temperature

The range in T_RUM_ was influenced by Day (*p* < 0.0001), Treatment × Day (*p* = 0.009), Phase (*p* < 0.0001), Phase × Day (*p* < 0.0001) and Treatment × Phase (*p* = 0.007). There were no effects of Treatment (*p* = 0.89) or Treatment × Phase × Day (*p* = 0.82). Mean range in T_RUM_ for Treatments was similar (*p* ≥ 0.36) during each of the five Phases (Table 3). The mean range in T_RUM_ of all Treatments decreased (*p* < 0.0001) during Phase III, with mean range in T_RUM_ being similar during Phase III and Phase IV (*p* ≥ 0.06; Table 3). The mean minimum range in T_RUM_ was on d 10 for T1 (0.40 ± 0.07 °C), d 12 for T2 (0.45 ± 0.07 °C) and d 11 for T3 (0.35 ± 0.07 °C). During Phase V, all Treatments presented a greater (*p* ≤ 0.01) mean range in T_RUM_ compared with Phase IV. Mean range in T_RUM_ were similar (*p* ≥ 0.07) for all Treatments during Phase V compared with during Phase I and Phase II.

#### 3.4.3. Duration of Rumen Temperature Above 42 °C

The T_RUM_ DUR > 42 °C was influenced by Day (*p* < 0.0001) and Phase (*p* < 0.0001), however there were no effects of Treatment (*p* = 0.69), Treatment × Day (*p* = 0.97), Treatment × Phase (*p* = 1.00), Phase × Day (*p* = 0.16) or Treatment × Phase × Day (*p* = 0.99). Steers in T2 only had DUR > 42 °C during Phase III (Figure 3). Mean DUR > 42 °C and AAC for all Treatments was greater during Phase III compared with Phase IV (*p* < 0.0001; Figure 3). Mean maximum T_RUM_ DUR > 42 °C and AAC was on d 9 for T1 (3.48 h/d, 1.03 AAC) and T2 (3.35 h/d, 1.03 AAC), and on d 11 for T3 (3.09 h/d, 1.34 AAC).

#### 3.4.4. Diurnal Rhythm of Rumen Temperature

During Phase I, the daily mean minimum T_RUM_ occurred at 1200 h, regardless of Treatment (T1, 39.40 ± 0.08 °C; T2, 39.48 ± 0.03 °C; T3, 39.36 ± 0.03 °C; Figure 3). Daily mean maximum T_RUM_ occurred at 9:00 pm for T1 (40.29 ± 0.08 °C) and T2 (40.34 ± 0.03 °C) and at 7:00 pm for T3 (40.32 ± 0.04 °C; Figure 3). During Phase II, diurnal T_RUM_ rhythm was similar to that recorded during Phase I, with daily mean minimum occurring between 10:00 am and 12:00 pm, and daily mean maximum occurring between 6:00 pm and 9:00 pm. During Phase III, the T_RUM_ diurnal rhythm was altered so that daily mean minimum occurred earlier in the morning at 10:00 am for T1 (40.51 ± 0.11 °C) and T2 (40.60 ± 0.11 °C), and at 9:00 am for T3 (40.52 ± 0.10 °C). Daily mean maximum T_RUM_ occurred at 8:00 pm for both T1 (41.68 ± 0.06 °C) and T2 (41.61 ± 0.08 °C), and at 5:00 pm for T3 (41.80 ± 0.07 °C). During Phase IV, the diurnal rhythm shifted where the daily mean minimum occurred at 5:00 am for all Treatments (T1, 40.06 ± 0.12 °C; T2, 39.86 ± 0.13 °C; T3, 39.90 ± 0.13 °C), and a daily mean maximum at 9:00 pm for T1 (40.95 ± 0.10 °C) and at 8:00 pm for T2 (40.79 ± 0.12 °C) and T3 (40.86 ± 0.12 °C). During Phase V, the T_RUM_ diurnal rhythm reached a daily mean minimum at 9:00 am, regardless of Treatment (T1, 38.66 ± 0.06 °C; T2, 38.64 ± 0.05 °C; T3, 38.61 ± 0.07 °C). The daily mean maximum occurred at 5:00 pm for T1 (39.62 ± 0.06 °C) and T2 (39.44 ± 0.05 °C), and at 9:00 pm for T3 (39.54 ± 0.05 °C).

### 3.5. Rumen pH

#### 3.5.1. Mean Rumen pH

**Figure 4:**
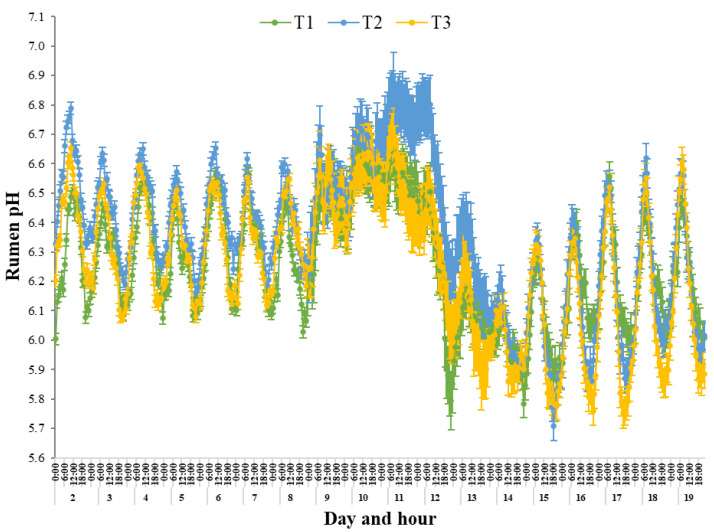
Mean (±SEM) rumen pH of steers when housed in climate control rooms for 21 days and split into five Phases consisting of pre-heat wave conditions (i) d 0–6, Phase I and; (ii) d 7–8, Phase II to reflect transition to heat load diet for T3 on d 7; acute heat wave conditions (iii) d 9–11, Phase III and (iv) d 12–13 Phase IV; and post-heat wave conditions (v) d 14–20 Phase V. Three dietary treatments were implemented: Treatment 1 (T1) was fed a finisher diet for the 21 days; Treatment 2 (T2) transitioned from the finisher diet to a heat load diet (heat load diet) on d 9 and fed the heat load diet until d 13; Treatment 3 (T3) transitioned from the finisher diet to the heat load diet on d 7 and fed the heat load diet until d 13. On d 14, T2 and T3 transitioned back to the finisher diet.

Mean rumen pH was influenced by Day (*p* < 0.0001), Phase (*p* < 0.0001), Phase × Day (*p* < 0.0001), Treatment × Phase (*p* < 0.0001) and Treatment × Phase × Day (*p* < 0.0001). There were no effects of Treatment (*p* = 0.19) or Treatment × Day (*p* = 0.51). Mean rumen pH for all Treatments was similar (*p* ≥ 0.12) during Phase I and Phase II, indicating that the transition to the heat load diet during Phase IV for T3 did not result in a change in mean rumen pH (Figure 4). Mean rumen pH of all Treatments increased (*p* < 0.0001) during Phase III from Phase II, increasing by 0.25, 0.26 and 0.18 pH units for T1, T2 and T3, respectively. During Phase III, mean rumen pH for all Treatments was the greatest (*p* < 0.0001), with mean rumen pH of T2 being greater (*p* = 0.04) compared with T3 and tended to be greater (*p* = 0.06) compared with T1. Mean rumen pH decreased (*p* < 0.0001) for all Treatments during Phase IV (T1, 0.40; T2, 0.32; T3, 0.39 pH unit decrease) from Phase III, with mean rumen pH being greater (*p* ≤ 0.006) for T2 compared with T1 and T3. Mean maximum rumen pH occurred on d 10 for T1 (6.56 ± 0.03) and T3 (6.55 ± 0.02) and on d 11 for T2 (6.76 ± 0.02). The mean minimum pH occurred on d 14 for all Treatments (T1, 5.99 ± 0.03; T2, 6.03 ± 0.02; T3, 5.97 ± 0.02). Mean rumen pH decreased (*p* < 0.0001) during Phase V for T2 (0.20 pH value; 3.17% decrease) and T3 (0.06 pH value; 1.01% decrease) but was no different (*p* = 0.74) for T1. During Phase V, mean rumen pH was the lowest (*p* < 0.0001) for Phase and was no different (*p* ≥ 0.21) across the three Treatments. Compared with Phase I and Phase II, mean rumen pH during Phase V for all Treatments was 0.14 to 0.27 pH units lower, and when compared with Phase III, mean rumen pH during Phase V was 0.39 to 0.52 pH units lower.

#### 3.5.2. Range in Rumen pH

The range in rumen pH was influenced by Day (*p* < 0.0001), Phase (*p* < 0.0001), Phase × Day (*p* < 0.0001), Treatment × Day (*p* < 0.0001), Treatment × Phase (*p* < 0.0001) and Treatment × Phase × Day (*p* < 0.0001). There were no effects of Treatment (*p* = 0.56). Mean range in rumen pH was similar (*p* ≥ 0.67) for Treatments during Phase I and Phase II (Table 4). During Phase III, the mean range in rumen pH decreased (*p* < 0.0001). During Phase IV, mean range in rumen pH was lower (*p* ≤ 0.009) for T2 compared with T1 and T3. The mean minimum range in rumen pH was on d 11 for all Treatments (T1, 0.14 ± 0.02; T2, 0.10 ± 0.02; T3, 0.11 ± 0.02). During Phase V, all Treatments presented a greater (*p* < 0.0001) mean range in rumen pH compared with during Phase III and Phase IV. During Phase V T2 and T3 had a lower (*p* < 0.0001) mean range in rumen pH, but were no different (*p* = 0.92) for T1, when compared with Phase I and Phase II.

#### 3.5.3. Duration of Rumen pH in SARA Thresholds

Mean DUR within SARA range was influenced by Day (*p* < 0.0001), Phase (*p* < 0.0001), Phase × Day (*p* < 0.0001), Treatment (*p* ≤ 0.006), Treatment × Phase (*p* < 0.0001) and Treatment × Phase × Day (*p* < 0.0001). There were no effects of Treatment × Day (*p* ≥ 0.78). During Phase IV, T3 had greater (*p* = 0.01) DUR within SARA and AUC compared with T2, but was similar to T1 (*p* = 0.17), with T1 and T2 also not different (*p* = 0.23; Table 5). The DUR within SARA and AUC was greatest (*p* ≤ 0.02) during Phase V for all Treatments, with T3 having the greatest (*p* ≤ 0.03) DUR within SARA compared with T1 and T2. Over the 21 days, T2 had the lowest (*p* ≤ 0.007) DUR within SARA. The mean maximum DUR in SARA and AUC was on d 14 for T1 (2.60 h/d, 2.22 AUC), and on d 15 for T2 (2.43 h/d, 0.82 AUC) and T3 (3.69 h/d, 1.17 AUC).

#### 3.5.4. Duration of Rumen pH Below ARA Thresholds

Mean DUR below ARA was influenced by Phase (*p* < 0.0001), Phase × Day (*p* < 0.0001), Treatment × Phase (*p* < 0.0001) and Treatment × Phase × Day (*p* < 0.0001). There were no effects of Treatments (*p* = 0.13), Day (*p* = 1.00), or Treatment × Day (*p* = 1.00). Steers in T1 and T2 had DUR below ARA during each Phase, whereas T3 only had DUR below ARA during Phase V (Table 6). However, T1 and T2 had very low DUR below ARA (≤ 0.03 h/d) during Phase I, Phase II and Phase III. During Phase IV, the DUR below ARA was greater (*p* < 0.0001) for T1 compared with T2 and T3, with no difference between T2 and T3 (*p* = 0.96). During Phase V, DUR below ARA was greater (*p* ≤ 0.05) for T1 compared with T2 and T3, with no difference between T2 and T3 (*p* = 0.82). The mean maximum DUR below ARA and AUC was on d 14 for T1 (0.55 h/d, 0.04 AUC) and T2 (0.005 h/d, 0.001 AUC), and on d 15 for T3 (0.08 h/d, 0.02 AUC).

#### 3.5.5. Diurnal Rhythm Rumen pH

During Phase I, the diurnal pH rhythm reached a daily mean minimum at 11:00 pm and 12:00 am for T1 (6.12 ± 0.04), at 11:00 pm for T2 (6.24 ± 0.03) and at 9:00 pm and 10:00 pm for T3 (6.13 ± 0.03). The daily mean maximum pH occurred at 0900 h for all Treatments (T1 6.47 ± 0.04; T2 6.64 ± 0.03; T3 6.55 ± 0.04; Figure 4). During Phase II diurnal pH rhythm was similar to that recorded during Phase I, with daily mean minimum occurring between 8:00 pm and 11:00 pm, and daily mean maximum occurring between 8:00 am and 9:00 am. There was a notable disruption of the diurnal pH rhythm for across all Treatments, at the onset of the heat wave conditions. On d 9, there was little evidence of a clear daily rhythm (Figure 4). During Phase III, the diurnal pH rhythm reached a daily mean minimum at 3:00 am for T1 (6.45 ± 0.08) and T3 (6.43 ± 0.04), with minimum pH being maintained between 7:00 pm and 8:00 pm for T2 (6.60 ± 0.06). The daily mean maximum occurred at 9:00 am for T1 (6.62 ± 0.08) and T2 (6.74 ± 0.05), and at 8:00 am and 9:00 am for T3 (6.62 ± 0.04). During Phase IV, rumen pH continued to lack a clear diurnal pH rhythm, whereas the daily mean minimum pH occurred between 10:00 pm and 1:00 am, and daily mean maximum occurred between 7:00 am and 9:00 am. A clear daily diurnal rhythm was evident by d 15, with the diurnal pH rhythm during Phase V being similar to Phase I and Phase II, reaching a daily mean minimum at 9:00 pm and 11:00 pm for T1 (5.97 ± 0.05), 8:00 pm for T2 (5.89 ± 0.03) and maintained between 6:00 pm and 9:00 pm for T3 (5.84 ± 0.03). The daily mean maximum occurred at 8:00 am, regardless of Treatments (T1 6.35 ± 0.05; T2 6.44 ± 0.04; T3 6.40 ± 0.05).

## 4. Discussion

Within the current study, a 66.07% to 77.67% reduction in DMILW was observed during the heat wave conditions, regardless of the dietary treatments provided, which is consistent with previous studies [26,27,28,29,30]. Kahl et al. [31] concluded that during periods of high heat load, decreases in feed intake are associated with the animal attempting to reduce metabolic heat production as a mechanism to maintain homeostasis. Within the current study, the mean DMILW of the T2 steers during Phase IV was comparatively lower when compared with the T3 steers and tended to be lower than T1 steers. The lower DMILW of T2 may be associated with the sudden dietary change to the heat load diet that occurred on d 9. This sudden change in diet may have prevented these steers from acclimating to the new diet, preventing ruminal adaptions before the onset of the thermal challenge, likely adding an additional stressor to the steers in T2. It is probable that this resulted in a rapid alteration in the rumen environment from the combination of dietary change in conjunction with the onset of heat wave conditions. Previously, Sejian et al. [32] evaluated the cumulative impact of multiple stressors (nutritional, thermal and locomotory) on the adaptive capability of rams. Outcomes from this study highlighted that the cumulative effect of these stressors negatively influenced feed intake, body temperature and general animal well-being, when compared with rams that had been exposed to a singular heat stressor.

Daily DMILW was greatly affected by the onset of Phase III, with feed consumption markedly decreasing until d 11, when mean minimum DMILW occurred for all Treatments. It is generally accepted that 3 to 4 days are needed after the onset of a heat event for cattle to begin to acclimatise and achieve a new level of heat balance [2]. At the onset of Phase IV, on d 12, DMILW began to increase for all Treatments, which coincided with a reduction in daily maximum ambient temperature by 4 °C, providing an opportunity to dissipate some accumulated heat load, as evidenced by decreases in mean T_RUM_ of between 0.47 and 0.69 °C. The severe decreases in DMILW, as well as exposure to the acute heat wave conditions, resulted in very low, although not unexpected, average daily gain. Despite the strong interaction of Treatment × Phase for DMILW, average daily gain was not affected by Treatments. Rhoads et al. [33] showed that decreased feed intake led to a favoured mobilisation of nutrients from peripheral tissues to compensate for subsequent increases in energy demands, rather than supporting anabolic and metabolic processors. This can partly explain why periods of heat stress are associated with decreases in average daily gain. Furthermore, typically, during heat wave events, there are large losses in body weight, which are associated with the decreased dry mater intake and the coinciding diversion of energy and nutrients away from growth and towards maintaining homeostasis [33,34]

Within the current study, the WILW for T1 and T3 tended to be greater during the heat wave conditions compared to pre-heat wave Phase I and Phase II, despite the heat load conditions increasing in intensity. It can be speculated that the mild heat load conditions during Phase I and Phase II may have influenced WILW, regardless of Treatments, so that WILW was already high due to the steers attempting to regulate their body temperature to facilitate evaporative heat loss and maintain homeostasis while exposed to chronic heat load conditions. This may in part be due to the dramatic decreases in DMILW negating the need for a greater water requirement for digestive processes. In comparison, the WILW for T2 clearly increased during the heat wave conditions compared with mean WILW pre-heat wave. Generally, water intake is primarily driven by dry matter intake and diet type, where previous studies have shown an increase in dry matter intake and diets with a higher roughage content are associated with an increase in water intake [35,36]. Despite this, the reasoning behind the comparatively greater WILW for T2 may be due to the direct effects of the heat wave conditions increasing water requirements for thermoregulatory mechanisms rather than being driven by dry matter intake and the greater roughage content of the heat load diet. Increased water intake during hot conditions can also be attributed to increases in water losses from peripheral vasodilation and evaporative cooling mechanisms, such as through increased respiration rate and panting, to regulate internal body temperature and the associated salivary losses that can occur with panting [37,38]. Water loss was not measured during this study; as such, the water lost between Treatments cannot be identified to verify the WILW differences. Furthermore, it is important to consider that on the respective d 7 and d 9 when T3 and T2 transitioned to the heat load diet, the DMILW and WILW of each Treatment was similar when compared with the previous day water intakes. This may indicate that the greater roughage content of the heat load diet did not influence the DMILW or WILW of the steers, possibly due to the roughage content only being increased by 8%, on an ingredient basis. Despite this, the greater WILW and lower DMILW of T2 during the heat wave conditions may be indicative of impaired thermoregulation in these animals, when compared with cattle in other Treatments and may not be due to the direct dietary effects.

Under thermoneutral conditions, it is generally considered that body temperature is regulated within a ± 1 °C gradient [39]. Within the current study, increases in T_RUM_, ranging between 0.8 and 1.01 °C, were observed for all Treatments when steers were transitioned from Phase II to the acute heat wave conditions during Phase III. Hahn [2] and Wahrmund et al. [40] suggested that disruptions to internal body temperature control during heat load periods can reflect thermal exchange imbalances between individual animals and the environment, as well as altered metabolic processors, activity level, feed and water intake levels and animal health status. The increased T_RUM_ within the current study may indicate that the thermoregulatory ability of these cattle became compromised during Phase III. Furthermore, there were no differences in T_RUM_ mean, range or DUR > 42 °C for Treatments, demonstrating that regulation of T_RUM_ was not influenced by the different dietary Treatments. Unsurprisingly, the T_RUM_ of all steers was influenced by Day and Phase, specifically during the acute heat wave conditions. The mean DUR > 42 °C and AAC increased, range in T_RUM_ decreased and the diurnal rhythm was altered. As such, it is likely that the acute heat wave conditions imposed during Phase III/Phase IV were likely to limit metabolic heat loss capabilities, as well as other heat dissipation thermoregulatory mechanisms, contributing to the greater heat load placed on these steers. This was evident as the proportion of time where T_RUM_ DUR > 42 °C was greatest during Phase III, with all steers having a T_RUM_ DUR > 42 °C for between 2.33 and 2.76 h per day. Previously, Mehla et al. [24] concluded that an internal body temperature of ≥ 42 °C may result in the homeostatic systems within the body reaching their upper critical limits for normal function. Therefore, changes in T^RUM^ surpassing this 42 °C threshold could be considered to be representative of a failure of the animal to adapt to the heat challenge imposed in this study.

It has been well established that body temperature is not static and, as such, exhibits a diurnal rhythm [41,42,43]. Variations in body temperature have been reported to range between 0.5 and 1.2 °C during moderate conditions, specifically, ambient temperatures of 30 ± 7 °C for tympanic temperature [2], 0.5 to 1.4 °C during thermoneutral to moderate conditions, consisting of ambient temperatures between 18 ± 7 °C and 34 ± 7 °C, for rectal temperature [26], and between 0.33 and 0.64 °C during heat wave conditions, where ambient temperature was > 35 °C, for T_RUM_ [44]. These previous findings suggest that the range in body temperature appears to be largely dependent on ambient conditions, although the location body temperature is measured as well as individual animal, managerial and environmental factors that appear to contribute to the variability that exists. Within the current study, mean range in T_RUM_ was similar for all Treatments during the pre- and post-heat wave conditions, indicating that T_RUM_ fluctuated each day, regardless of Treatments. In comparison, during the heat wave conditions of Phase III/Phase IV, the range in T_RUM_ for all Treatments decreased. Furthermore, Hahn and Mader [3] suggested that during heat load events, where ambient temperature was 32 ± 7 °C, the diurnal rhythm of body temperature becomes disrupted with peaks in maximum body temperature, typically lagging ambient conditions by 3 to 5 h. Similar lag periods were observed within the current study, with mean maximum T_RUM_ lagging ambient conditions by 4 to 7 h from when maximum heat load was imposed at 1300 h. There was also a clear alteration in daily diurnal rhythm during the heat wave where mean T_RUM_ minimum and maximum values occurred around 2 h earlier during the morning and around 2 h earlier during the late evening, respectively, irrespective of Treatment. However, these changes to body temperature rhythm may be confounded by the climate-controlled environment where natural outdoor conditions may support the thermal exchange pathways more efficiently when compared to chamber housing. Regardless, the altered rhythm in daily body temperature observed in this study is similar to previous heat load studies [26,41,42,45,46]. Furthermore, these changes can be considered a reflection of heat accumulation and dissipation during heat load conditions. Lefcourt and Adams [41] reported that this shift to increasing body heat dissipation during the earlier morning period could be attributed to a physiological reset mechanism that cattle employ in preparation for the onset of continued heat challenge with the commencement of a new day. Within the current study, despite thermal conditions cooling during Phase V, the T_RUM_ diurnal rhythm did not return to pre-heat wave rhythms, as recorded during Phase I and Phase II. This is in agreement with a previous study by Hahn and Mader [3] and, more recently, Wijffels et al. [29] and Sammes et al. [30]. Wijffels et al. [29], determined that this post-heat-wave reduced body temperature is an allostatic response to the heat challenge. This results in body temperature adjustments that stabilise around a new lower temperature, which drives new adjusted physiological state post-heat event [29].

Within the current study, all Treatments had the greatest mean rumen pH during the first 3 days of the heat wave, which were during Phase III, compared with pre- and post-heat wave conditions, possibly associated with the very low DMILW. Generally, during periods of nutrient deprivation, the growth of some rumen microorganisms is inhibited, thus reductions in acid production can occur, resulting in an increase in rumen pH for extended periods [47]. Furthermore, when a dual-flow, continuous-culture system was used to investigate effects of normal T_RUM_ (39 °C) and high T_RUM_ (41 °C) on in vitro fermentative conditions, the high T_RUM_ conditions increased (*p* < 0.01) rumen pH [48]. Conversely, in a study by Mishra et al. [49], higher concentrations of lactic acid and lower rumen pH values were recorded for lactating dairy cows during heat stress (ambient temperature, 29.4 °C; relative humidity, 85%), which may be attributed to the feeding of high concentrate diets. Overall, this suggests that these altered concentrations of lactic acid and pH might be involved in inhibiting rumen motility with a negative influence on rumen health [49]. A study by Crossland et al. [50] reported that mean rumen pH of eight crossbred finishing steers (389 ± 30 kg) was lower (*p* = 0.0029) during hot (ambient temperature, 35 ± 0.55 °C; relative humidity, 42%) compared with thermoneutral (ambient temperature, 18 ± 0.55 °C; relative humidity, 20%) conditions, although there was no difference in pH variation (*p* = 0.3328). The authors speculated that during heat stress a slower rumen passage rate and decreased acid clearance within the rumen may be associated with the lower pH [50]. Within the current study, the greater rumen pH of all Treatments could be attributed to the acute heat wave conditions, high T_RUM_, increased WILW and low DMILW of the steers.

Within the current study, the T2 steers maintained comparatively greater rumen pH values during the heat wave conditions compared with T1 and T3. The tendencies for lower DMILW and greater WILW of T2 during the heat wave conditions is sufficient to suggest that these steers had greater rumen pH due to these altered feed and water intakes, when compared to the similar intake responses of T1 and T3. Interestingly, the rumen pH of T2 increased beginning from d 10, maintaining comparatively elevated pH values separated from the similar daily rumen pH trend, as seen for T1 and T3 up until d 13. Upon evaluation, the T2 steers maintained, on average, a rumen pH 0.21 times greater than the other Treatments during the heat wave, reaching a maximum divergence of mean daily pH on d 11, of which was 0.24 times greater compared with T1 and T3. The greater rumen pH recorded for T2 may indicate impaired rumen functionality due to the tendencies for lower DMILW and greater WILW diluting ruminal contents during the heat wave. This may suggest the dysregulation of rumen pH, WILW and DMILW separate from the trends seen for T1 and T3 may possibly be due to T2 transitioning to the heat load diet on the first day of Phase III, compounding the impact of heat stress. It is well known that the establishment of stable rumen microbial populations during dietary transition is not immediate [51]. It is probable that the direct effects of the acute heat load conditions negatively impacted the ability of the steers to maintain metabolic stability and was confounded by the sudden dietary change and concurrent suppression of DMILW and increase in WILW. Despite this, the T2 steers spent a lower DUR within SARA and below ARA compared with T1. This may suggest that despite a possible compound effect of dietary transition coinciding with onset of the heat wave conditions, the greater roughage concentration of the heat load diet may be responsible for the reduced DUR below the ARA threshold for both T2 and T3 when compared with T1. A greater proportion of roughage in the diet can result in longer chewing times and greater saliva production, and thus bicarbonate buffer entering the rumen, which neutralises and dilutes ruminal acids, helping to reduce the risk of acidosis [52,53]. In the current study, rumen pH of all Treatments sharply declined 4 days after the onset of the heat wave conditions, which coincided with a reduction in mean T_RUM_, an increase in DMILW after 3 days of near-ceased DMILW and decrease in heat load conditions. Generally, at the conclusion of heat wave conditions cattle generally compensate for low feed intake during the heat wave, typically via excessive consumption of feed. This excessive consumption of concentrates can result in accumulation of acid in the rumen, which lowers rumen pH and can contribute to causing a range of acidotic and metabolic problems [17]. As a consequence of the acute heat wave conditions and subsequent 3 days of high T_RUM_ and nutrient deprivation from very low DMILW, followed by an increase in DMILW, this may have altered rumen function, negatively affected regulation of rumen pH and increased DUR spent below acidosis thresholds. Findings within the current study suggest that during the post-heat wave period of Phase V, the steers were unable to return to rumen pH conditions similar to the pre-heat wave conditions. This may suggest that irrespective of dietary Treatment, the imposed acute heat wave conditions may have negatively altered the ruminal environment, disrupting the functionality and regulation of rumen pH, as seen with the sharp decline in mean rumen pH during Phase IV. These disturbances to rumen pH appear to then remain and intensify during the post-heat wave period, despite DMILW increasing and T_RUM_ decreasing.

During Phase I and Phase II, the rumen pH was elevated during mid-morning, reaching a daily maximum between 8:00 am and 9:00 am and a daily minimum during the late evening, between 8:00 pm and 12:00 am for all Treatments. There was a notable disruption of the diurnal pH rhythm across all Treatments at the onset of the heat load conditions on d 9, whereas there was little evidence of a clear daily rhythm during Phase III and Phase IV. This may have been due to greater mean rumen pH, mean T_RUM_ and WILW, reduced range in rumen pH and low DMILW during the heat wave conditions. Despite this, the times of the day that rumen pH reached a daily minimum and maximum were similar to that recorded pre-heat wave, being elevated during mid-morning, between 0800 h and 0900 h, and a nadir in the late evening, although this was over a slightly extended time period between 7:00 pm and 3:00 am. Salfer et al. [54] found that although differences in daily mean rumen pH were evident for different diets, including differing concentrations of starch, fatty acids and dietary fibre, there was no difference in the daily diurnal pattern of rumen pH. This is in agreement with the current study in that, unlike the T_RUM_ diurnal rhythm that became greatly disrupted in terms of when daily minimums and maximums occurred during each Phase, the daily rumen pH diurnal rhythm remained relatively unchanged throughout each Phase. Furthermore, the current study found there to be no difference in mean rumen pH between T1 and T3 during any Phase. Such data suggests that despite being fed different diets, this did not influence rumen pH; however, this may be due to the very low DMILW of the steers during the heat wave negating any dietary influence. In comparison, T2 maintained comparatively greater mean rumen pH during the heat wave conditions of which was particularly evident during Phase IV. The greater rumen pH of T2 during the heat wave may be associated with the sudden dietary change to the heat load diet on d 9, which tended to reduce DMILW and increase WILW compared with T1 and T3. From d 14, all Treatments evidently regained a clear pH diurnal rhythm within the preceding Phase V days as T_RUM_ and WILW decreased, and DMILW and range in rumen pH gradually increased.

During Phase V, all Treatment had the greatest time spent below SARA and ARA thresholds for Phase. Interestingly, steers in T2 had the lowest DUR within SARA range and below the ARA threshold, possibly due to the tendency for lower DMILW and greater WILW resulting in greater mean rumen pH during the heat wave. In comparison, steers in T3 spent the greatest DUR within SARA range during Phase IV and Phase V, with T1 having the greatest DUR below ARA range during Phase IV and Phase V, possibly due to the lower proportion of roughage in the finisher diet. The increased roughage content of the heat load diet may explain why the steers in both T2 and T3 spent a lower DUR below the ARA threshold. A greater proportion of roughage in the diet has been shown to result in longer chewing times and greater saliva production, thus bicarbonate entering the rumen, which neutralises and dilutes ruminal acids, helping to reduce the risk of acidosis [52,53]. Beatty et al. [27] reported that after a hot period, cattle are not able to maintain homeostasis and metabolic disorders can develop, possibly due to inappetence during prolonged high heat load periods. In addition, Bernabucci et al. [55] suggested that when high heat load conditions are removed and animals normalise their physiological response parameters and feed intakes, the functionality of the rumen environment remains impaired for 1 to 2 weeks. Furthermore, Crossland et al. [50] reported that T_RUM_ greatly affects rumen pH and the duration and magnitude of SARA and ARA, possibly due to altered metabolism. Within the current study, the acute heat wave conditions were associated with a substantial variation in the rumen environment; when rumen temperature became elevated, the range in rumen pH decreased and a clear deviation from diurnal rhythm in rumen pH ceased. These ruminal disturbances may have negatively influenced regulation of rumen pH during the preceding 7 days into Phase V. This may partly explain why the greatest DUR spent below acidosis thresholds occurred during Phase V and is sufficient to suggest that acidosis in grain-fed beef steers appears to occur predominately during post-heat wave periods rather than during heat wave conditions, therefore suggesting that feeding a heat load diet during the post-heat wave period may address these ruminal disturbances and increased DUR spent below the ARA threshold. Within the current study, the increased roughage content of the heat load diet may have helped regulate rumen pH so that the occurrence of ARA was reduced for T2 and T3, which may improve animal performance in the long term. This is an area that warrants further investigation under large-scale commercial feedlot conditions to elucidate heat load diet timing efficacy in grain-fed steers during heat wave conditions. However, the foundational outcome from this study is that further work focusing on implementing feeding strategies during post-heat wave periods to support rumen recovery is vital.

## 5. Conclusions

The heat load conditions imposed on these cattle were sufficient to elicit heat load responses, allowing the effectiveness of the three feeding strategies to be evaluated. All cattle experienced significant physiological changes, as indicated by alterations in DMILW, T_RUM_, rumen pH and diurnal rhythms, during exposure to the acute heat wave conditions. Steers in T1 spent the greatest DUR below the ARA threshold during Phase IV and Phase V compared with T2 and T3, possibly due to the lower proportion of roughage in the finisher diet. The increase in the DUR within SARA range and below ARA threshold occurred 4 days after the onset of the acute heat wave conditions, which coincided with a reduction in the imposed heat load conditions, decrease in mean T_RUM_ and increase in DMILW. It is important to consider impact on cattle in T2; the results presented here highlighted the compounding negative impacts from a sudden diet change that coincided with the commencement of the heat challenge.

Overall, the outcomes from this study highlight that altering the feeding management practices of grain-fed beef cattle is a viable option to help regulate the rumen environment during periods of heat load. Furthermore, the outcomes of this study have shown that feeding strategies to manage the rumen environment may be more important to implement post-heat wave periods rather than prior to or during heat wave conditions.

## Figures and Tables

**Table 1:** Diet and nutrient composition of each diet.

Item	Starter	Intermediate	Finisher	Heat Load
Ingredient				
Sub-batch grain mix ^1^	62.1	74.5	86.8	78.7
Whole cottonseed	9.0	16.5	9.0	9.0
Lucerne hay	28.9	9.0	4.2	12.3
Nutrient composition, DM				
Dry Matter, %	88.0	89.3	88.7	88.6
Acid Detergent Fibre, ADF, %	26.3	25.7	11.9	17.7
Neutral Detergent Fibre, %	40.4	37.5	22.9	25.3
Net Energy for Gain, MJ/kg	2.9	2.9	3.0	3.0
Crude Fat, %	4.6	4.3	4.6	4.3
Crude Protein, %	17.3	15.8	15.1	15.1
Crude Fibre, %	21.8	19.7	8.7	12.4
Nitrogen-Free Extract, %	50.3	54.8	67.8	68.5
Metabolizable Energy, MJ/kg ^2^	11.6	11.9	13.2	13.1
Digestible Energy, MJ/kg	14.3	14.7	16.3	16.2
Feed Digestibility, %	76.8	79.1	86.1	86.8
Digestible Dry Matter, %	67.6	70.7	76.3	76.9
Digestible Protein, %	13.3	12.5	13.0	13.1
Starch, %	22.9	21.8	43.2	43.2

^1^ Sub-batch grain mix: feedlot pellet 9.2%, steam-rolled barley 89.2%, vegetable oil 1.6%. Feedlot pellet: millrun wheat 55.9%, ammonium sulphate 2.6%, dry-rolled wheat 12.5%, calcium carbonate (limestone) 15.6%, Rumensin 100 0.3%, magnesium oxide 0.7%, Availa zinc 100 0.34%, vegetable oil 3.1%, salt, plain (NaCl) 2.8%, urea 5.7%, vitamin A 500 0.009%, vitamin E 0.057%, XFE-Select L 0.385. ^2^ ME (MJ/kg DM) = 0.12 × CP + 0.31 × EE + 0.05 × CF + 0.14 × NFE.

**Table 2:** Effects of treatments on mean (± SEM) dry matter intake (DMI) expressed as a percentage (%) of live weight (DMILW) of grain-fed beef steers when housed in climate control rooms.

Item	Phase ^1^	Treatments ^2^			SEM
		T1	T2	T3	
Mean DMILW, % LW/steer/d	Phase I	1.74	1.82	1.85	0.06
	Phase II	1.74	1.89	1.89	0.07
	Phase III	0.59	0.42	0.58	0.06
	Phase IV	0.76	0.60	0.81	0.07
	Phase V	1.36	1.34	1.41	0.06

^1^ Days were split into five Phases for comparison of animal data based on transition to diets and climate schedule, consisting of mild heat load conditions (i) d 0–6, Phase I and (ii) d 7–8, Phase II; acute heat wave conditions (iii) d 9–11, Phase III and (iv) d 12–13 Phase IV; and mild heat load conditions (v) d 14–20 Phase V. ^2^ Three dietary treatments were implemented: Treatment 1 (T1) was fed a finisher diet for the 21 days; Treatment 2 (T2) transitioned from the finisher diet to a heat load diet (heat load diet) on d 9 and fed the heat load diet until d 13; Treatment 3 (T3) transitioned from the finisher diet to the heat load diet on d 7 and fed the heat load diet until d 13. On d 14, T2 and T3 transitioned back to the finisher diet.

**Table 3:** Effects of treatments on mean rumen temperature (T_RUM_) range of grain-fed beef steers when housed in climate control rooms.

Item	Phase ^1^	Treatments ^2^			SEM
		T1	T2	T3	
T_RUM_ Range, °C	Phase I	0.54	0.58	0.62	0.06
	Phase II	0.58	0.63	0.61	0.06
	Phase III	0.42	0.49	0.41	0.06
	Phase IV	0.48	0.46	0.45	0.06
	Phase V	0.57	0.62	0.59	0.06

^1^ Days were split into five Phases for comparison of animal data based on transition to diets and climate schedule, consisting of mild heat load conditions (i) d 0–6, Phase I and (ii) d 7–8, Phase II; acute heat wave conditions (iii) d 9–11, Phase III and (iv) d 12–13 Phase IV; and mild heat load conditions (v) d 14–20 Phase V. ^2^ Three dietary treatments were implemented: Treatment 1 (T1) was fed a finisher diet for the 21 days; Treatment 2 (T2) transitioned from the finisher diet to a heat load diet (heat load diet) on d 9 and fed the heat load diet until d 13; Treatment 3 (T3) transitioned from the finisher diet to the heat load diet on d 7 and fed the heat load diet until d 13. On d 14, T2 and T3 transitioned back to the finisher diet.

**Table 4:** Effects of treatments on mean rumen pH of grain-fed beef steers when housed in climate control rooms.

Item	Phase ^1^	Treatments ^2^			SEM
		T1	T2	T3	
rumen pH range	Phase I	0.21	0.21	0.20	0.01
	Phase II	0.22	0.22	0.22	0.01
	Phase III	0.15	0.14	0.14	0.01
	Phase IV	0.17 ^ac^	0.11 ^b^	0.16 ^ac^	0.01
	Phase V	0.21	0.18	0.18	0.01

^1^ Days were split into five Phases for comparison of animal data based on transition to diets and climate schedule, consisting of mild heat load conditions (i) d 0–6, Phase I and (ii) d 7–8, Phase II; acute heat wave conditions (iii) d 9–11, Phase III and (iv) d 12–13 Phase IV; and mild heat load conditions (v) d 14–20 Phase V. ^2^ Three dietary treatments were implemented: Treatment 1 (T1) was fed a finisher diet for the 21 days; Treatment 2 (T2) transitioned from the finisher diet to a heat load diet (heat load diet) on d 9 and fed the heat load diet until d 13; Treatment 3 (T3) transitioned from the finisher diet to the heat load diet on d 7 and fed the heat load diet until d 13. On d 14, T2 and T3 transitioned back to the finisher diet.

**Table 5:** Effects of treatments on the duration of time rumen pH was within sub-acute acidosis (SARA) thresholds of grain-fed beef steers when housed in climate control rooms.

Item	Phase ^1^	Treatments ^2^			SEM
		T1	T2	T3	
rumen pH range	Phase I	0.70	---	0.67	0.50
	Phase II	1.10	---	0.41	0.49
	Phase III	0.68	---	0.13	0.49
	Phase IV	1.25 ^bc^	0.47 ^b^	2.23 ^c^	0.50
	Phase V	1.69 ^ab^	1.26 ^ab^	3.20 ^c^	0.49

^1.^ Days were split into five Phases for comparison of animal data based on transition to diets and climate schedule, consisting of mild heat load conditions (i) d 0–6, Phase I and (ii) d 7–8, Phase II; acute heat wave conditions (iii) d 9–11, Phase III and (iv) d 12–13 Phase IV; and mild heat load conditions (v) d 14–20 Phase V. ^2^ Three dietary treatments were implemented: Treatment 1 (T1) was fed a finisher diet for the 21 days; Treatment 2 (T2) transitioned from the finisher diet to a heat load diet (heat load diet) on d 9 and fed the heat load diet until d 13; Treatment 3 (T3) transitioned from the finisher diet to the heat load diet on d 7 and fed the heat load diet until d 13. On d 14, T2 and T3 transitioned back to the finisher diet.

**Table 6:** Effects of treatments on the duration of time rumen pH was below acute acidosis (ARA) thresholds of grain-fed beef steers when housed in climate control rooms.

Item	Phase ^1^	Treatments ^2^			SEM
		T1	T2	T3	
rumen pH range	Phase I	0.03	0.002	---	0.09
	Phase II	0.03	0.002	---	0.10
	Phase III	0.02	0.002	---	0.09
	Phase IV	0.67 ^a^	0.002 ^bc^	--- ^bc^	0.10
	Phase V	0.34 ^a^	0.004 ^bc^	0.057 ^bc^	0.09

^1^ Days were split into five Phases for comparison of animal data based on transition to diets and climate schedule, consisting of mild heat load conditions (i) d 0–6, Phase I and (ii) d 7–8, Phase II; acute heat wave conditions (iii) d 9–11, Phase III and (iv) d 12–13 Phase IV; and mild heat load conditions (v) d 14–20 Phase V. ^2^ Three dietary treatments were implemented: Treatment 1 (T1) was fed a finisher diet for the 21 days; Treatment 2 (T2) transitioned from the finisher diet to a heat load diet (heat load diet) on d 9 and fed the heat load diet until d 13; Treatment 3 (T3) transitioned from the finisher diet to the heat load diet on d 7 and fed the heat load diet until d 13. On d 14, T2 and T3 transitioned back to the finisher diet.

## Data Availability

Data will be made available on reasonable request from the corresponding author.

## Abbreviations

The following abbreviations are used in this manuscript:

T_RUM_	Rumen temperature, °C
DMILW	Dry matter intake as a percentage of live weight
WILW	Water intake as a percentage of live weight
THI	Temperature Humidity Index
T1	Treatment 1, control diet where cattle were fed a finisher diet for the 21 days
T2	Treatment 2, cattle transitioned from the finisher diet to a HLD on d 9 and fed the heat load diet until d 14
T3	Treatment 3, cattle transitioned from the finisher diet to the heat load diet on d 7 and fed the heat load diet until d 14
